# Mortality after cardiopulmonary resuscitation on a medical ICU: A sex-specific outcome analysis

**DOI:** 10.1007/s00508-021-01831-0

**Published:** 2021-03-09

**Authors:** Richard Rezar, Bernhard Wernly, Michael Haslinger, Clemens Seelmaier, Philipp Schwaiger, Ingrid Pretsch, Maria Eisl, Christian Jung, Uta C. Hoppe, Michael Lichtenauer

**Affiliations:** 1 Clinic of Internal Medicine II, Department of Cardiology, Paracelsus Medical University of Salzburg, 5020 Salzburg, Austria; 2 Division of Cardiology, Pulmonology and Vascular Medicine, Medical Faculty, Heinrich-Heine-University, Düsseldorf, Germany

**Keywords:** CPR, Resuscitation, Sex, Outcome, Cardiac arrest

## Abstract

**Background:**

Performing cardiopulmonary resuscitation (CPR) and postresuscitation care in the intensive care unit (ICU) are standardized procedures; however, there is evidence suggesting sex-dependent differences in clinical management and outcome variables after cardiac arrest (CA).

**Methods:**

A prospective analysis of patients who were hospitalized at a medical ICU after CPR between December 2018 and March 2020 was conducted. Exclusion criteria were age < 18 years, hospital length of stay < 24 h and traumatic CA. The primary study endpoint was mortality after 6 months and the secondary endpoint neurological outcome assessed by cerebral performance category (CPC). Differences between groups were calculated by using U‑tests and χ^2^-tests, for survival analysis both univariate and multivariable Cox regression were fitted.

**Results:**

A total of 106 patients were included and the majority were male (71.7%). No statistically significant difference regarding 6‑month mortality between sexes could be shown (hazard risk, HR 0.68, 95% confidence interval, CI 0.35–1.34; *p* = 0.27). Neurological outcome was also similar between both groups (CPC 1 88% in both sexes after 6 months; *p* = 1.000). There were no statistically significant differences regarding general characteristics, pre-existing diseases, as well as the majority of clinical and laboratory parameters or measures performed on the ICU.

**Conclusion:**

In a single center CPR database no statistically significant sex-specific differences regarding post-resuscitation care, survival and neurological outcome after 6 months were observed.

## Introduction

Medical differences between women and men are sometimes obvious, sometimes subtle. Especially in the case of cardiac arrest (CA), one would assume that “symptoms of death are incontrovertible”, as Helviz et al. once stated [1]. Furthermore, it is interesting that especially after a perfectly standardized procedure, such as cardiopulmonary resuscitation (CPR), significant differences regarding outcome were observed in various studies in the past [1]. Different authors have examined epidemiological, directly procedure-related, as well as outcome parameters and not all of them are in line. In many studies male individuals are younger when facing cardiac arrest, show initially shockable rhythms more often and tend to suffer an arrest in public places more frequently, whereas a difference regarding bystander CPR cases could not be observed by all authors [1–5]. It was also shown that women are less likely to receive guideline-compliant CPR and face differences in postresuscitation care, such as targeted temperature management (TTM) or percutaneous coronary interventions (PCI) [2, 3]. Regarding outcome, contradictory results were observed. Some authors described a better outcome in female patients, while others reported reverse findings [1]. With respect to neurological outcome, in some studies indifferent results were observed but there was a trend towards a worse outcome for female patients [1]. Whether it is due to women often facing cardiac arrest at home alone, initial electrocardiography (ECG) findings differ between men and women or if general pathophysiological differences exist, need to be determined. We aimed to analyze sex-associated differences in a contemporary CPR cohort to further advance understanding of past findings and help to find possible approaches for a better outcome regardless of sex in the future.

## Methods

### Study subjects

Patients who were hospitalized at the intensive care unit (ICU, Division of Cardiology, Paracelsus Medical University Salzburg) after cardiopulmonary resuscitation between December 2018 and March 2020 were included in the prospective CPR database. Exclusion criteria were age under 18 years, hospital length of stay (LOS) of less than 24 h and traumatic cardiac arrest. The study was conducted according to the principles of the Declaration of Helsinki and good clinical practice. A positive vote from the local ethics committee was obtained (415-E/2408/8-2018). Patients were included after obtaining informed consent if possible. Individuals with poor neurological outcome or patients who died without being verbally responsive were included depending on the presumed will with the help of the next of kin if possible. Follow-up examinations by telephone after 30 days and 6 months included neurologic assessment via cerebral performance category (CPC).

### Statistical analysis

The studyʼs primary endpoint was all-cause mortality after 6 months, the secondary endpoint was neurological outcome assessed by CPC. The first four categories of the CPC score were used (1: good cerebral performance/normal life; 2: moderate cerebral disability/disabled but independent; 3: severe cerebral disability/conscious but disabled and dependent; 4: coma/vegetative state/unconscious) and patients who died within 6 months were classified as deceased. Individuals with certified brain death after 1 or 6 months (CPC 5) but with preserved other organ functions were not observed in this cohort. Statistical analyses were performed using Stata/IC 16.1 for Mac (64-bit Intel; StataCorp LLC, College Station, TX, USA). Patient baseline characteristics, comorbidities, medical measures, laboratory values and outcomes were analyzed for the overall cohort. Categorical variables are given as numbers and percentages, continuous variables, data as median ± interquartile range (IQR). Differences between male and female individuals were calculated using U‑test and χ^2^-test. Cox regression analysis was used for evaluation of associations with the endpoint. Hazard ratios (HR) as well as adjusted hazard ratios (aHR) with 95% confidence intervals (CI) were calculated. A multivariable Cox regression model was performed. As there were 32 events (deaths), the amount of covariables was restricted based on the “one in ten rule” to avoid overfitting [6]. The covariables age, SOFA (Sequential Organ Failure Assessment) score and lactate concentration at admission were included based on clinical relevance and the multivariable model built using the forced entry method. *P*-values were considered statistically significant if < 0.05, all tests were two-sided.

## Results

### General characteristics

A total of 106 patients were included in our study, median patient age was 65 years for both cohorts and no difference was found regarding body mass index (BMI; male (M): 26.1 kg/m^2^ vs. female (F): 25.7 kg/m^2^; *p* = 0.735). Male individuals made up a large proportion of the total patient population (71.7%; *n* = 76 vs. 30). Rates of out-of-hospital cardiac arrest did not differ statistically significantly between male and female individuals (M: 86.8% vs. F: 80%; *p* = 0.380) and no statistically significant difference regarding occurrence of shockable rhythms was found (M: 77.6% vs. F: 60.0%; *p* = 0.272). Bystander CPR was performed statistically significant more often in female patients (100% vs. 75.9%, *p* = 0.02). No difference concerning prior medical history (arterial hypertension, sleep apnea, coronary artery disease, cardiac surgery, chronic obstructive pulmonary disease) was observed. Other cardiovascular illnesses and history of smoking were also evenly distributed (see Table 1). Regarding cause of cardiac arrest, no difference was observed for acute coronary syndrome (M: 60.5% vs. F: 63.3%), but a trend towards a higher incidence of primarily rhythmological events (23.7% vs. 3.3%) in men was shown. Respiratory failure (13.3% vs. 5.3%) and pulmonary embolism (10.0% vs. 3.9%) were numerically more common in female individuals. No difference regarding SOFA score (sequential organ failure assessment; median in both groups 11 points; *p* = 0.400) and simplified acute physiology score (SAPS) II, was observed (M: 78 points vs. F: 80.5 points; *p* = 0.269). No statistically significant difference was found for mechanical ventilation, duration of ventilation, use of vasopressors, targeted temperature management, continuous renal replacement therapy, antimicrobial therapy, systemic lysis, blood transfusion or PCI (see Table 1). Overall left ventricular ejection fraction also did not differ (see Table 1).

**Table 1:** Baseline characteristics of male and female patients

Characteristic	Male (*n* = 76)	Female (*n* = 30)	*p*-value
*Age—years—median (IQR)*	65 (53–74)	65 (57–75)	0.763
*BMI—kg/m*^*2*^*—median (IQR)*	26.1 (24.5–29.0)	25.7 (24.1–29.1)	0.735
*OHCA—no. (%)*	66 (86.8)	24 (80.0)	0.380
*Initial rhythm—no. (%)*	–	–	0.272
Shockable	59 (77.6)	18 (60.0)	–
Non-shockable	16 (21.1)	10 (33.3)	–
Unknown	1 (1.3)	2 (6.7)	–
*Bystander CPR—no (%)*	44 (75.9)	20 (100)	**0.02**
Unknown	18 (23.7)	10 (33.3)	–
*Admission to TCC—no (%)*	–	–	0.651
Direct	59 (77.6)	24 (80.0)	–
Interfacility	12 (15.8)	3 (10.0)	–
Intrahospital TCC	5 (6.6)	3 (10.0)	–
*Transport modality—no (%)*	–	–	0.555
Airborne	27 (35.5)	13 (43.3)	–
Ground transport	44 (57.9)	14 (46.7)	–
Intrahospital	5 (6.6)	3 (10.0)	–
*Smoker—no (%)*	36 (47.4)	14 (46.7)	0.205
*Comorbidities—no. (%)*	–	–	–
Arterial hypertension	53 (69.7)	18 (60.0)	0.625
Diabetes mellitus	14 (18.4)	5 (16.7)	0.776
Hyperlipidemia	41 (53.9)	16 (53.3)	0.789
CKD	15 (19.7)	5 (16.7)	0.758
COPD	8 (10.5)	5 (16.7)	0.511
OSA	6 (7.9)	1 (3.3)	0.670
History of CAD	15 (19.7)	3 (10.0)	0.268
History of cardiac surgery	7 (9.2)	1 (3.3)	0.432
*Cause of cardiac arrest—no. (%)*	–	–	0.117
ACS	46 (60.5)	19 (63.3)	–
Rhythmological	18 (23.7)	1 (3.3)	–
Asphyxia/respiratory failure	4 (5.3)	4 (13.3)	–
Pulmonary embolism	3 (3.9)	3 (10.0)	–
Other	5 (6.6)	3 (10.0)	–
*SOFA score at admission—median (IQR)*	11 (9–12)	11 (9–12)	0.400
*SAPS II at admission—median (IQR)*	78 (70.5–84.8)	80.5 (75–85.8)	0.269
*Mechanical ventilation—no. (%)*	73 (96.1)	27 (90.0)	0.348
*Duration of mechanical ventilation—h—median (IQR)*	48.5 (34–133)	43 (24–143)	0.636
*Delirium—no (%)*	20 (26.3)	4 (13.3)	0.200
*Vasopressor use—no. (%)*	70 (92.1)	28 (93.3)	> 0.99
*Targeted temperature management—no. (%)*	53 (69.7)	20 (66.7)	0.817
*Continuous hemodiafiltration—no. (%)*	8 (10.5)	2 (6.7)	0.721
*Antimicrobial therapy—no. (%)*	71 (93.4)	27 (90.0)	0.685
*Systemic lysis—no. (%)*	6 (7.9)	3 (10.0)	0.710
*Blood transfusion—no. (%)*	10 (13.2)	3 (10.0)	0.755
*Coronary angiography—no. (%)*	60 (78.9)	21 (70.0)	0.324
*PCI—no. (%)*	42 (55.3)	17 (56.7)	> 0.99
*Left ventricular ejection fraction—no. (%)*	–	–	> 0.99
Normal	11 (14.5)	6 (20.0)	–
Mildly abnormal	17 (22.4)	8 (26.7)	–
Moderately abnormal	27 (35.5)	8 (26.7)	–
Severely abnormal	21 (27.6)	8 (26.7)	–

*ACS* acute coronary syndrome,* BMI* body mass index,* CAD* coronary artery disease, *CKD* chronic kidney disease, *COPD* chronic obstructive pulmonary disease,* hr* hours,* ICU* intensive care unit,* IQR* interquartile range,* OHCA* out of hospital cardiac arrest,* OSA* obstructive sleep apnea,* PCI* percutaneous coronary intervention, *PEA* pulseless electrical activity,* SAPS II* simplified acute physiology score II,* SOFA* sequential organ failure assessment,* TCC* tertiary care center

Regarding laboratory measures, no difference regarding initial pH, lactate and base excess values (BE) was observed (pH: M: 7.210 vs. F: 7.251, *p* = 0.543; lactate: M: 2.6. vs. F: 2.5, *p* = 0.822; BE: M: −8.0 mmol/L vs. F: −9.4 mmol/L; *p* = 0.797). Blood glucose at admission was significantly higher in female patients (13 mmol/L vs. 8.9 mmol/L; *p* = 0.001), albeit no difference was found in median glycosylated hemoglobin (HbA1c, F: 37.7 mmol/mol vs. M: 36.6 mmol/mol; *p* = 0.052). Female individuals showed statistically significant lower hemoglobin values (14.3 mg/dL vs. 12.9 mg/dL; *p* = 0.001), similar leucocyte (M: 14.3 G/L vs. F: 14.8 G/L; *p* = 0.449) and thrombocyte counts (M: 225 G/L vs. F: 273 G/L; *p* = 0.041). Levels of C‑reactive protein, serum electrolytes, blood urea, liver functional parameters and low-density lipoprotein did not differ (see Table 2). In males, higher serum creatinine levels were observed (1.3 mg/dL vs. 1.1 mg/dL; *p* = 0.005). No difference was found regarding initial creatine kinase (M: 224 U/L vs. F: 192 U/L; *p* = 0.483) and high-sensitive troponin T (M: 148 ng/L vs. F: 112 ng/L; *p* = 0.720).

**Table 2:** Laboratory values of male and female patients

Lab value	Male	Female	*p*-value
pH-value at admission—median (IQR)	7.210 (7.091–7.308)	7.251 (6.995–7.363)	0.543
Lactate at admission—mmol/L—median (IQR)	2.6 (1.7–4.5)	2.5 (1.6–5.0)	0.822
Base excess at admission—mmol/L—median (IQR)	−8.0 (−13.6 to −5.3)	−9.4 (−14.0 to −5.3)	0.797
Blood glucose at admission—mmol/L—median (IQR)	8.9 (7.7–12)	13 (10–16.1)	**0.001**
Hemoglobin at admission—g/dL—median (IQR)	14.3 (13.2–15.2)	12.9 (12.3–13.5)	**0.001**
Leucocyte count at admission—G/L—median (IQR)	14.3 (10.6–17.8)	14.8 (12.7–18.4)	0.449
Thrombocyte count at admission—G/L—median (IQR)	225 (187–273)	273 (205–329)	0.041
CRP at admission—mg/dL—median (IQR)	0.3 (0.1–1.0)	0.5 (0.3–1.6)	0.080
Sodium at admission—mmol/L—median (IQR)	139 (136–141)	138 (136–139)	0.229
Chloride at admission—mmol/L—median (IQR)	100 (97–102)	100 (98–101)	0.688
Calcium at admission—mmol/L—median (IQR)	2.2 (2.1–2.3)	2.2 (2.2–2.3)	0.278
Potassium at admission—mmol/L—median (IQR)	4 (3.6–4.6)	3.8 (3.6–4.3)	0.343
Creatinine at admission—mg/dL—median (IQR)	1.3 (1.1–1.4)	1.1 (0.9–1.3)	**0.005**
Urea at admission—mg/dL—median (IQR)	39 (33–49)	38 (31–45)	0.410
AST—U/l—median (IQR)	150 (93–258)	253 (95–535)	0.098
ALT—U/l—median (IQR)	111 (63–219)	166 (59–305)	0.168
Bilirubin—mg/dL—median (IQR)	0.5 (0.4–0.8)	0.4 (0.3–0.6)	0.048
GGT at admission—U/L—median (IQR)	59 (41–106)	44 (30–71)	0.054
CHE—U/mL—median (IQR)	5.9 (7.0–7.9)	7.0 (6.5–8.4)	0.502
Albumin—g/dL—median (IQR)	3.5 (3.0–4.0)	3.0 (3.0)	0.683
Creatine kinase at admission—U/L—median (IQR)	224 (147–614)	192 (121–545)	0.483
hsTroponin T—ng/L—median (IQR)	148 (51–447)	112 (47–708)	0.720
HbA1c—mmol/mol—median (IQR)	36.6 (34.4–41)	37.7 (35.5–39.9)	0.052
HbA1c not available—no (%)	13 (17.1)	10 (33.3)	–
LDL—mg/dL—median (IQR)	72 (51–106)	77 (46–108)	0.92
LDL not available—no (%)	4 (5.3)	1 (3.3)	–

*ALT* alanine aminotransferase,* AST* aspartate aminotransferase,* CHE* cholinesterase,* CRP C-*reactive protein, *GGT* gamma-glutamyltransferase,* hsTroponin T* high-sensitive troponin T,* IQR* interquartile range,* LDL* low-density lipoprotein

### Outcome and survival analysis

**Fig. 1:**
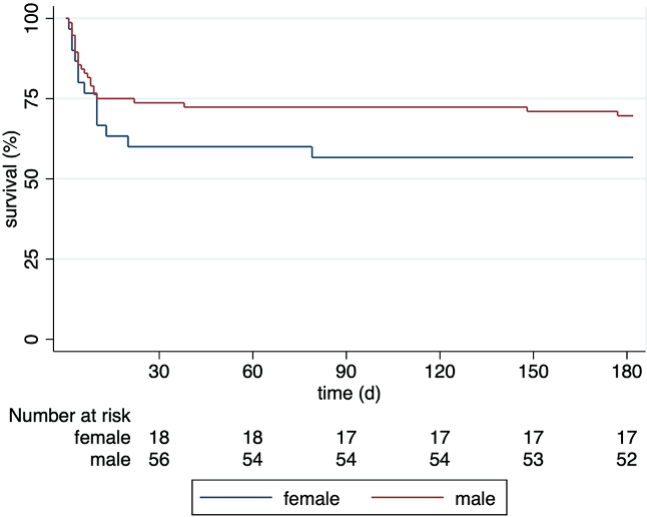
Kaplan-Meier plot for 6‑month survival. The mortality was similar in male and female patients (HR 0.68, 95%CI 0.35–1.34; *p* = 0.27). *d* days

No difference was found regarding ICU length of stay (M: 128 hours vs. F: 114 hours; *p* = 0.594), as well as overall hospital stay (M: 9 days vs. F: 7 days; *p* = 0.168), albeit duration of hospitalization could not be obtained in some patients as they were transferred to other hospitals. No statistically significant difference was found for occurrence of delirium (M: 26.3% vs. F: 13.3%; *p* = 0.200). Regarding neurological outcome, no difference was observed after 1 and 6 months (Table 3). Survival after 6 months was numerically higher in males (68% vs. 56.7%), although the difference was not (HR 0.68 95%CI 0.35–1.34; *p* = 0.27) statistically significant. This finding persisted in sensitivity analyses evaluating patients above 65 years (M: 43% vs. F: 50%; *p* = 0.76) and below 65 years (M: 12% vs. F: 31%; *p* = 0.12), patients with an initial lactate concentration ≥ 2.5 mmol/L (M: 46% vs. F: 64%; *p* = 0.35) and below 2.5 mmol/L (M: 6% vs. F: 20%; *p* = 0.14), an initial left ventricular ejection fraction < 30% (M: 14% vs. F: 38%; *p* = 0.31) and ≥ 30% (M: 31% vs. F: 41%; *p* = 0.43), patients with (M: 15% vs. F: 32%; *p* = 0.18) and without (M: 43% vs. F: 55%; *p* = 0.73) underlying acute coronary syndrome (ACS) and an initially shockable (M: 17% vs. F: 28%; *p* = 0.32) and non-shockable rhythm (M: 59% vs. F: 58%; *p* > 0.99). Also, the mortality remained similar between sexes after multivariable adjustment (aHR 1.65, 95%CI 0.80–3.38; *p* = 0.17). A plotted Kaplan-Meier survival curve is provided in Fig. 1.

**Table 3:** Outcome of male and female patients

Outcome	Male	Female	*p*-value
ICU length of stay—hr—median (IQR)	128 (69–220)	114 (51–235)	0.594
*Hospital length of stay—days median (IQR)*	–	–	0.168
Days—median (IQR)	9 (5–20)	7 (4–12)	–
Transferred to other hospital—no. (%)	15 (19.7)	9 (30.0)	–
*Survival at 30 days—no (%)*	56 (73.7)	18 (60.0)	0.167
*Survival at 6 months—no (%)*	51 (68.0)	17 (56.7)	0.272
*Neurological outcome at 30 days—no (%)*	–	–	0.630
CPC 1	44 (78.6)	13 (72.2)	–
CPC 2	6 (10.7)	2 (11.1)	–
CPC 3	3 (5.4)	2 (11.1)	–
CPC 4	3 (5.4)	1 (5.6)	–
*Neurological outcome at 6 months—no (%)*	–	–	> 0.99
CPC 1	45 (88.2)	15 (88.2)	–
CPC 2	6 (11.8)	2 (11.8)	–

*CPC* cerebral performance category,* hr* hours, *ICU* intensive care unit, *IQR* interquartile range

## Discussion

**Fig. 2:**
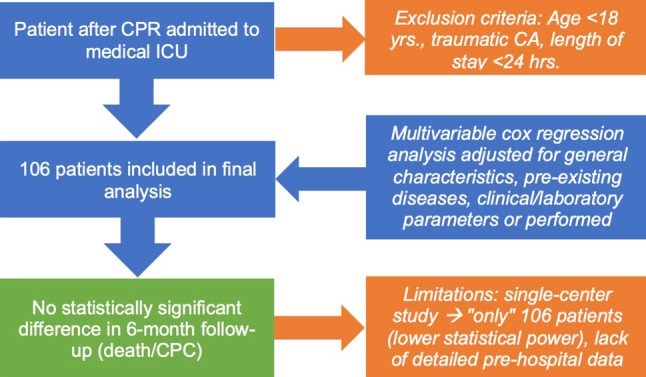
Graphical abstract. *CA* cardiac arrest,* CPC* cerebral performance category,* hrs.* hours, *ICU* intensive care unit,* yrs*. years

In this exploratory real-world register including 106 consecutive patients admitted to a medical ICU from December 2018 to March 2020 no statistically significant sex-specific differences regarding mortality and neurological outcome after 6 months could be shown. A graphical abstract of the study is provided in Fig. 2.

Sex differences represent a crucial health topic both in general medicine as well as in the acute care setting. Previous studies, including large registries, found inconsistent rates of different clinical and outcome variables between men and women after CA [4, 7–11]. We aimed to further investigate possible differences in clinical course, survival and neurological outcome between sexes in an Austrian CPR cohort.

A total of 106 individuals with nontraumatic CA were admitted to our medical ICU. Baseline characteristics, including age, BMI and comorbidities were similar between sexes. Unlike other trials which showed survival rates in the period from hospital admission to discharge of as low as 10% [4], we found a higher rate of survivors at 6 months follow-up (~65%). This may be (partly) explained by excluding patients who died within 24 h of admission. Among the patients in this study, no statistically significant sex-associated differences were found regarding the primary endpoint all-cause mortality and neurological outcome as a secondary endpoint. Comparable mortality rates after 1 and 6 months were consistent after multivariable adjustment. Statistically insignificant but numerical differences (e.g. rhythmological cause of CA, mortality, occurrence of delirium) could possibly become relevant in a larger study cohort, although the study endpoints remained similar in a multivariable regression analysis. In the literature dissimilar results regarding outcome were observed in the past. Blewer et al. for example [10] analyzed a US American and Canadian CPR database with more than 19,000 OHCA patients and found significantly increased odds of survival for men compared to women (OR 1.29, CI 1.17–1.42). In contrast to our study, all OHCA patients were analyzed, regardless of survival at admission to the hospital and therefore including those who died at the scene.

Other trials, like a Danish registry study [7] with more than 19,000 patients over a period of 10 years found benefits favoring women over men. Female patients were more likely to survive in cases of a primarily shockable rhythm but showed equal outcomes in non-shockable rhythms; however, there are major differences to consider. The baseline data showed higher survival of men, which inverted after adjusting for patient-related and cardiac arrest-related characteristics. Only 13.2% of the patients survived until hospital arrival. As only the latter, relatively small group of patients was included in our study, this underlines the heterogeneity of different patient collectives in the general literature. Nevertheless, we could observe numerically higher but still statistically insignificant rates of initially shockable rhythms in male individuals and a larger number of men suffered from a primary rhythmological cause of arrest, whereas women were more likely to face CA due to respiratory causes and pulmonary embolism.

Interestingly, an age-related difference regarding outcome could not be observed in our patient collective. In contrast, previous registry trials reported more older female patients facing CA, whereas this age difference was also associated with higher mortality [7, 8]. Older, postmenopausal females were previously described to be at higher risk compared to men of comparable age, which could be due to possible post-ischemic cardiac mechanisms of female endogenous estrogen and progesterone hormones [4, 12]. Our results do not contribute to this hypothesis, as the subgroup analysis of women below and over 65 years showed similar mortality rates compared to age-matched men. Another important factor regarding resuscitation is the application of bystander CPR, which greatly improves survival [10, 11]. In previous studies it was shown that women are less likely to face witnessed CA and/or receive bystander CPR [7, 8, 10]. We could not confirm these results since in our study, women received bystander CPR more often, although no data regarding performance of bystander CPR were available for 25% of all patients.

In our study, female individuals had markedly higher blood sugar levels on admission, yet equal HbA1c-counts when compared to men. Cofactors, such as body mass index (BMI), diabetes mellitus and age were similar between both groups. One possible explanation could be that women certainly receive relatively higher doses of adrenaline during prehospital advanced life support (per m^2^ body surface area or kg body weight). Since catecholamines trigger gluconeogenesis and to a certain extent a dose-dependent effect exists [13, 14], this could be a possible explanation. Unfortunately, no data on received cumulative catecholamine doses were available for our patient collective. In our cohort, women weighed less (median 73 vs. 80.5 kg) and had a decreased body surface area (median 1.84 vs. 2.00 m^2^) compared to men, but assumably received the same absolute catecholamine doses on the scene as resuscitation protocols usually do not distinguish between sexes. The definitive cause of this finding remains unclear and should be subject to further investigation. We could also observe a marked difference in hemoglobin and serum creatinine levels between the sexes, whereas men had higher values of both parameters. The differences regarding hemoglobin and creatinine have been known for a long time [15, 16] and widely implemented into clinical practice by different thresholds.

Overall, the diverse findings of this and previous trials accentuate the lack of a complete understanding of all contributing factors to the crucial improvement of the chain of survival after cardiac arrest. More research with special focus on sex-specific differences is warranted.

### Limitations

The limitations of this study are its single-center character and the relatively small number of patients. Numerically a difference regarding mortality could be shown, although not statistically significant, probably due to a lack of statistical power. One disadvantage is the lack of sufficient prehospital data like dispatch-to-CPR intervals, duration of CPR, number of shocks, cumulative drug doses and time-to-hospital. We only included patients admitted to the ICU, which represents a rather small portion of all patients who suffer CA. We also present a mixed cohort of OHCA and IHCA patients. Even if we could not show a difference regarding outcome between OHCA and IHCA patients, this could be the case in a larger patient cohort. On the other hand, hereby we can provide real world data from the ICU-clinician’s point of view.

## Conclusion

Women and men showed similar clinical risk distribution, management strategies and mortality rates as well as comparable neurological outcome data after cardiac arrest, with a trend towards higher survival in men. Due to a low sample size, findings of this study should be considered hypothesis-generating and must be confirmed in larger studies.
